# Core–Double-Shell TiO_2_@Fe_3_O_4_@C Microspheres with Enhanced Cycling Performance as Anode Materials for Lithium-Ion Batteries

**DOI:** 10.3390/ma17112543

**Published:** 2024-05-24

**Authors:** Yuan Chen, Jiatong Yang, Aoxiong He, Jian Li, Weiliang Ma, Marie-Christine Record, Pascal Boulet, Juan Wang, Jan-Michael Albina

**Affiliations:** 1 Hubei Provincial Key Laboratory of Green Materials for Light Industry, Collaborative Innovation Center of Green Light-Weight Materials and Processing, School of Materials and Chemical Engineering, Hubei University of Technology, Wuhan 430068, China; 2 New Materials and Green Manufacturing Talent Introduction and Innovation Demonstration Base, Wuhan 430068, China; 3 Aix-Marseille University, IM2NP, CEDEX 20, 13397 Marseille, France; 4 CNRS, IM2NP, CEDEX 20, 13397 Marseille, France

**Keywords:** lithium-ion batteries, anode materials, carbon coating, core–double-shell structure, electrochemical properties

## Abstract

Due to the volume expansion effect during charge and discharge processes, the application of transition metal oxide anode materials in lithium-ion batteries is limited. Composite materials and carbon coating are often considered feasible improvement methods. In this study, three types of TiO_2_@Fe_3_O_4_@C microspheres with a core–double-shell structure, namely TFCS (TiO_2_@Fe_3_O_4_@C with 0.0119 g PVP), TFCM (TiO_2_@Fe_3_O_4_@C with 0.0238 g PVP), and TFCL (TiO_2_@Fe_3_O_4_@C with 0.0476 g PVP), were prepared using PVP (polyvinylpyrrolidone) as the carbon source through homogeneous precipitation and high-temperature carbonization methods. After 500 cycles at a current density of 2 C, the specific capacities of these three microspheres are all higher than that of TiO_2_@Fe_2_O_3_ with significantly improved cycling stability. Among them, TFCM exhibits the highest specific capacity of 328.3 mAh·g^−1^, which was attributed to the amorphous carbon layer effectively mitigating the capacity decay caused by the volume expansion of iron oxide during charge and discharge processes. Additionally, the carbon coating layer enhances the electrical conductivity of the TiO_2_@Fe_3_O_4_@C materials, thereby improving their rate performance. Within the range of 100 to 1600 mA·g^−1^, the capacity retention rates for TiO_2_@Fe_2_O_3_, TFCS, TFCM, and TFCL are 27.2%, 35.2%, 35.9%, and 36.9%, respectively. This study provides insights into the development of new lithium-ion battery anode materials based on Ti and Fe oxides with the abundance and environmental friendliness of iron, titanium, and carbon resources in TiO_2_@Fe_3_O_4_@C microsphere anode materials, making this strategy potentially applicable.

## 1. Introduction

Since the dawn of the 21st century, the rapid expansion of the new energy vehicle industry and the escalating demand for electronic products have propelled the widespread application of lithium-ion batteries [1,2]. As application scenarios increasingly demand higher battery performance [3], the efficacy of anode materials has become a pivotal factor affecting the energy storage efficiency, cycle life, charging and discharging rates, and safety of lithium-ion batteries. Consequently, developing anode materials with high energy density, high electrical conductivity, and high stability has emerged as a crucial research topic in the field [4].

Transition metal oxides, such as TiO_2_ [5,6], Fe_2_O_3_ [7], Fe_3_O_4_ [8], Co_3_O_4_ [9], MnO_2_ [10], CuO [11], NiO [12], etc., have emerged as promising anode materials for lithium-ion batteries due to their abundant reserves, low cost, environmental friendliness, high energy density, and excellent safety performance [13]. However, their widespread application is limited by issues such as low electrical conductivity, capacity loss due to irreversible reactions, and volume changes during cycling [14,15,16]. To address these issues, researchers have conducted numerous studies on the morphological and structural modifications of transition metal oxides, which are now generally focused on two main strategies: constructing nanostructures and material composites [17,18]. For instance, Kim et al. [19] successfully prepared nFe_3_O_4_@TiO_2_ through a sol–gel reaction involving TEOT and magnetite clusters, demonstrating superior capacity retention. Fan et al. [20] deposited Co_3_O_4_ on TiO_2_ nanotubes via a photo-deposition method, and the resulting Co_3_O_4_/TiO_2_ nanotube composite material exhibited better capacity and rate performance than pure TiO_2_ nanotubes. In a previous study, our group uniformly coated Fe_2_O_3_ on homemade TiO_2_ microspheres with good monodispersity to produce TiO_2_@ Fe_2_O_3_ microspheres with lychee-like core–shell structure, which exhibited higher lithium storage capacity and electrical conductivity due to the synergistic effect of TiO_2_ and Fe_2_O_3_ [21].

Carbon materials are a favorable choice for composites with transition metal oxides due to their excellent electrical conductivity and structural stability. Currently, novel composite anode materials have been developed by combining carbon-based materials with different transition metal oxides, demonstrating superior performance [22,23,24]. Tu et al. [25] employed a one-step spray pyrolysis strategy to prepare Fe_3_O_4_@C materials with pomegranate configuration, which exhibited excellent lithium-ion storage performance. Li et al. [26] prepared mesoporous hollow carbon@MnO_2_ nanospheres using lignosulfonate as a carbon source, showing exceptional cycling and rate performance. Therefore, we conjecture that if the TiO_2_@Fe_2_O_3_ microsphere material we have prepared continues to be composited with carbon, it may further enhance its electrochemical properties.

Based on the above ideas, in this study, TiO_2_@Fe_2_O_3_ microspheres were first prepared by coating a layer of Fe_2_O_3_ on the surface of homemade TiO_2_ microspheres using the homogeneous precipitation method. PVP molecules were then adsorbed onto the surface of the microspheres and subsequently transformed into carbon layers through high-temperature carbonization. During this process, some of the Fe^3+^ was reduced to Fe^2+^, resulting in the formation of TiO_2_@Fe_3_O_4_@C microspheres with a core–double-shell structure, and the effect of the amount of carbon coating on the electrochemical performance of these microspheres was studied. This study provides insight into the modification and carbon-coating strategy of anode materials for lithium-ion batteries. Additionally, the abundance and environmental friendliness of iron, titanium, and carbon resources make this strategy potentially valuable for practical applications.

## 2. Materials and Methods

### 2.1. Materials

The raw materials consisted of urea (CH_4_N_2_O, Sinopharm Chemical Reagent Co., Shanghai, China, AR), anhydrous ethanol (C_2_H_5_OH, Sinopharm Chemical Reagent Co., Shanghai, China, AR), FeCl_3_·6H_2_O (Aladdin Reagent Co., Shanghai, China, ACS), Fe_2_O_3_ (Heng Xing Reagent Co., Tianjin, China, AR), tetrabutyl titanate (TBOT, Shanghai Macklin Biochemical Co., Shanghai, China, AR), commercial graphite (C, Tianjin Battery Co., Tianjin, China, AR), and polyvinylpyrrolidone (PVP, Aladdin Reagent Co., Shanghai, China, AR). Solutions were prepared with deionized water from a Molecular Lab ultra-purifier (Shanghai, China).

The precursor TiO_2_ mesoporous microspheres used in this experiment are prepared using a low-temperature modified Stöber method, which has been previously detailed by our research group [27]. In this method, TBOT is used as the titanium source, and anhydrous C_2_H_5_OH serves as the reaction solvent. Initially, 2 mL KCl aqueous solution and 400 mL of anhydrous C_2_H_5_OH are added to a glass low-temperature reaction vessel. The mixture is then cooled and stirred for 1 h. Once the temperature drops to −10 °C, TBOT is quickly added and stirred for 5 min to ensure thorough mixing. The mixture is then left to stand for 5 h at low temperature. After this period, the precipitate is collected via centrifugation, washed three times each with anhydrous ethanol and deionized water, and then freeze-dried to obtain amorphous precursor TiO_2_ mesoporous microspheres.

### 2.2. Synthesis of TiO_2_@Fe_3_O_4_@C Microspheres with Core–Double-Shell Structure

**Figure 1:**
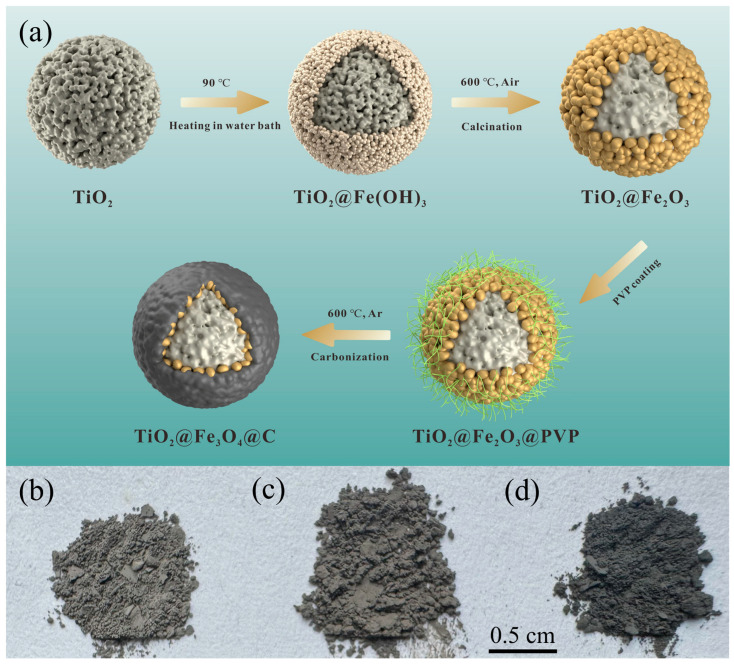
(**a**) Preparation schematic of TiO_2_@Fe_3_O_4_@C microspheres and digital images of (**b**) TFCS, (**c**) TFCM, (**d**) TFCL.

Figure 1a shows a schematic diagram of the preparation process of core–double-shell structured TiO_2_@Fe_3_O_4_@C microspheres. The preparation process is divided into two steps as follows.

The first step involves the preparation of core–shell structured TiO_2_@Fe_2_O_3_ microspheres. The TiO_2_@Fe_2_O_3_ microspheres were fabricated by the homogeneous precipitation method, which has been previously reported by our group [21]. To begin, 0.16 g of the self-prepared, well-dispersed TiO_2_ microspheres, 0.2705 g of FeCl_3_·6H_2_O, and 200 mL of deionized water were combined in a beaker, which was followed by ultrasonic dispersion for 20 min. Subsequently, 0.2424 g of urea was added to the solution, which was then stirred magnetically for 30 min. The beaker was then placed in a 90 °C water bath for heating and stirring, continuing for 3 h. Afterward, the reaction product was centrifuged and rinsed three times with deionized water, followed by lyophilization to obtain TiO_2_@Fe(OH)_3_ microspheres, which were calcined at 600 °C for 4 h to obtain TiO_2_@Fe_2_O_3_ microspheres.

The second step is the preparation of TiO_2_@Fe_3_O_4_@C microspheres with core–double-shell structures by coating a carbon layer on the surface of TiO_2_@Fe_2_O_3_ microspheres. A solution was prepared by dissolving a certain amount of polyvinylpyrrolidone (PVP) in 30 mL of anhydrous ethanol, into which 0.5 g of TiO_2_@Fe_2_O_3_ microspheres was added. This mixture was then ultrasonically dispersed for 20 min at room temperature, followed by magnetic stirring for 4 h, resulting in TiO_2_@Fe_2_O_3_@PVP microspheres. The resultant product was heated in a 78 °C water bath with stirring until all the anhydrous ethanol had evaporated. The obtained solid was then dried in a convection oven at 80 °C for 24 h, after which it was placed in a tube furnace and carbonized under an argon atmosphere at 600 °C for 4 h, yielding TiO_2_@Fe_3_O_4_@C microspheres. Keeping other conditions constant, we set the PVP additions at 0.0119 g, 0.0238 g, and 0.0476 g, and we named the final TiO_2_@Fe_3_O_4_@C microsphere products as TFCS, TFCM, and TFCL, respectively.

### 2.3. Structure Analysis and Characterization

The structural properties of the products were examined by X-ray diffraction (XRD) using a PANalytical Empyrean X-ray diffractometer (Almelo, Netherlands) with Cu Kα radiation (λ = 0.154 nm). The 2θ scan range was set from 20° to 90° at a scanning rate of 2°/min. Raman spectra were collected using a HORIBA XploRA PLUS spectrometer (Lille, France) with a 532 nm laser source. X-ray photoelectron spectroscopy (XPS) measurements were conducted using a PHI5000 VersaProbe III instrument (Kanagawa, Japan) equipped with a monochromated Al Kα X-ray source and achieving an energy resolution of less than 0.50 eV (Ag 3d 5/2). The sample morphologies and elemental ratios were analyzed using a SU8010 Hitachi field emission scanning electron microscope (FE-SEM, Tokyo, Japan) operating at 5 kV, paired with an energy dispersive X-ray spectrometer (EDS, X-MaxN, OXFORD, Oxford, UK).

### 2.4. Electrochemical Tests

The prepared microsphere anode materials, cotinine black (Shanghai Macklin Biochemical Co., Shanghai, China, AR) and polyvinylidene fluoride (PVDF, Shanghai Macklin Biochemical Co., Shanghai, China, AR), were weighed in a ratio of 7:2:1. These components were thoroughly combined and placed in a beaker containing N-Methylpyrrolidone (NMP, Shanghai Aladdin Biochemical Co., Shanghai, China, AR), and the mixture was magnetically stirred for 7 h. The resulting slurry was then coated onto 20 µm thick copper foil, completely dried at 80 °C, and subsequently cut into 15 mm diameter discs. A 1 M solution of LiPF6 in ethylene carbonate (EC), dimethyl carbonate (DMC), and ethyl methyl carbonate (EMC) (in a volume ratio of 1:1:1) was used as the electrolyte. A polypropylene film (Celgard 2500, Celgard Inc., Charlotte, NC, USA) was used as the separator, and lithium foil served as both the counter and reference electrode. The assembly of CR2032 half-cells was performed in an argon-filled glove box. The electrodes had a mass loading of around 1.0–1.4 mg cm^−2^. The battery test system (CT2001A, Land Corp., Ltd., Wuhan, China) was employed to assess the charge/discharge cycling performance and rate capability of the anode materials within a voltage range of 0.01 to 3.0 V (vs. Li/Li^+^). Cyclic voltammetry (CV) was carried out using an electrochemical workstation (CS350M, Corrtest Instrument Corp., Ltd., Wuhan, China) with a voltage window of 0.01 to 3.5 V and a scan rate of 0.1 mV·s^−1^. Electrochemical impedance spectroscopy (EIS) measurements were taken on the same device, covering frequencies from 1 × 10^6^ to 0.01 Hz.

## 3. Results and Discussion

### 3.1. Structural Analysis

**Figure 2:**
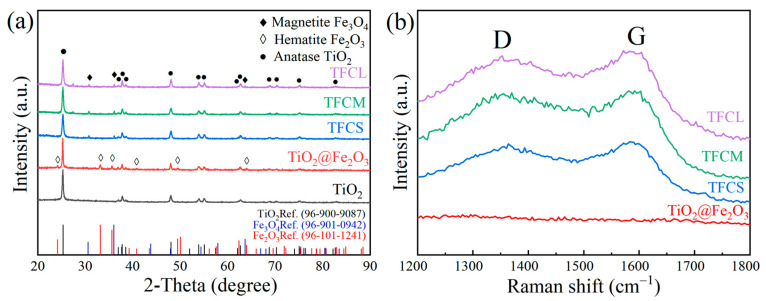
(**a**) XRD patterns of TiO_2_, TiO_2_@Fe_2_O_3_, TFCS, TFCM, TFCL (**b**) Raman spectra of TiO_2_, TiO_2_@Fe_2_O_3_, TFCS, TFCM, TFCL.

Figure 2a displays the X-ray diffraction patterns of mesoporous TiO_2_ microspheres, TiO_2_@Fe_2_O_3_ microspheres, and TiO_2_@Fe_3_O_4_@C microspheres with three different amounts of PVP added after calcination at 600 °C. It can be observed from the figure that all X-ray diffraction peaks of the calcined mesoporous TiO_2_ microspheres correspond to the anatase phase of TiO_2_ in the tetragonal crystal system (Ref. code: 96-900-9087), indicating that their structure is of the anatase phase. All X-ray diffraction peaks of the TiO_2_@Fe_2_O_3_ microspheres correspond to both the hematite α-Fe_2_O_3_ in the hexagonal crystal system (Ref. code: 96-101-1241) and the anatase TiO_2_ in the tetragonal crystal system (Ref. code: 96-900-9087). The X-ray diffraction peaks of the TiO_2_@Fe_3_O_4_@C microspheres with three different PVP additions are essentially identical, with diffraction peaks appearing at 2θ of 25.3°, 37.8°, 48°, 53.9°, 55.1°, 62.7°, and 75.2° corresponding to the anatase phase of TiO_2_ in the tetragonal crystal system (Ref. code: 96-900-9087), and peaks appearing at 2θ of 30.6°, 36°, and 63.6° corresponding to the magnetite Fe_3_O_4_ in the cubic crystal system (Ref. code: 96-901-0942). In the X-ray diffraction patterns of the TiO_2_@Fe_3_O_4_@C microspheres with three different amounts of PVP added, no diffraction peaks corresponding to the characteristic peaks of Fe_2_O_3_ were observed. This suggests that the relatively low content of Fe_2_O_3_ in the TiO_2_@Fe_2_O_3_ microspheres was completely reduced to Fe_3_O_4_ during the carbonization process [21]. Furthermore, no characteristic peaks related to carbon were present in the XRD results. Figure 1b–d depict the visual representation of the TiO_2_@Fe_3_O_4_@C microspheres with varying amounts of PVP added. The photos reveal that the surfaces of the microspheres appear gray or black, implying the presence of a carbon layer coating on their surface. Based on the XRD results, we may infer that the carbon layer generated from the high-temperature carbonization of the coated PVP is probably amorphous [28].

In order to further determine the structure of the carbon layer coated on the surface of TiO_2_@Fe_3_O_4_@C microspheres, we carried out Raman spectroscopy on TiO_2_@Fe_3_O_4_@C microspheres with three different PVP additions and TiO_2_@Fe_2_O_3_ microspheres, the results of which are shown in Figure 2b. Compared to TiO_2_@Fe_2_O_3_ microspheres, the defect-induced D band and the graphitic crystallite-derived G band in TiO_2_@Fe_3_O_4_@C microspheres have been detected at around 1351 and 1580 cm^−1^ [29]. The degree of disorder of the carbon material can be evaluated by calculating the ratio of peak intensities between two peaks, ID/IG. The larger the value of ID/IG, the higher the degree of disorder of the material and the more defects [30]. The calculated intensity ratios of ID/IG for TFCS (0.98), TFCM (0.97) and TFCL (0.97) remain high and almost equal, further demonstrating that the coated carbon layer on the surface of TiO_2_@Fe_3_O_4_@C microspheres keeps the amorphous feature, which is conducive to enhancing conductivity [31,32].

**Figure 3:**
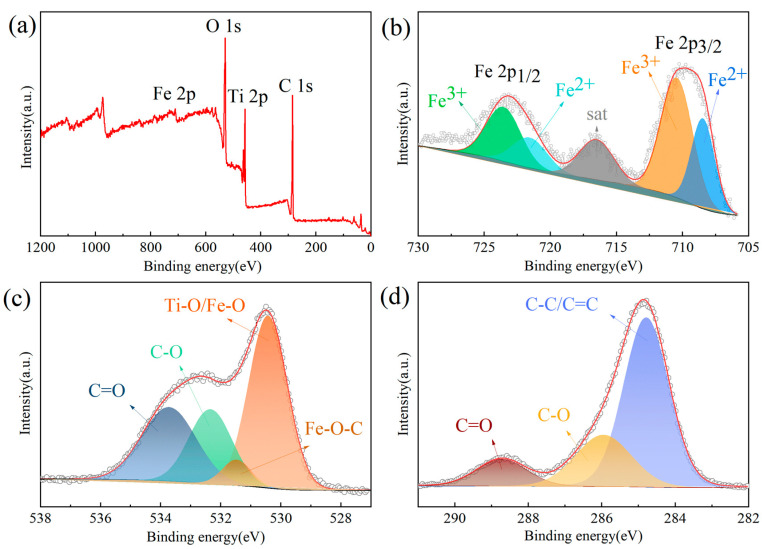
XPS spectra of TFCM (**a**) survey, (**b**) Fe 2p, (**c**) O 1s, (**d**) C 1s.

Figure 3a–d present the X-ray photoelectron spectroscopy (XPS) analysis results of TFCM. It should be noted that we chose TFCM as the subject for XPS testing for the following two reasons: First, among the three materials, TFCS, TFCM, and TFCL, TFCM exhibited superior electrochemical performance (as analyzed later), making its structural characteristics representative. Second, the Fe content in the three samples is equal and very low. According to the XRD results, all Fe_2_O_3_ in the three samples was reduced to Fe_3_O_4_. Therefore, the main difference among these three materials lies in the amount of coated carbon. Regarding the states of Fe, C, and O in these three materials, the XPS analysis results of TFCM should be representative. Hence, we chose TFCM as the subject for XPS testing. Figure 3a depicts the comprehensive XPS spectrum of TFCM, revealing the detection of four elements: Fe, O, Ti, and C, corroborating the findings from XRD and Raman spectroscopy. The high-resolution XPS spectrum of Fe 2p, as shown in Figure 3b, exhibits two broad peaks at 723.5 and 709.8 eV, corresponding to the characteristic peaks of Fe 2p_1/2_ and Fe 2p_3/2_, respectively. The broad peak at 723.5 eV can be fitted into the peaks at 723.7 eV and 721.7 eV, while the peak at 709.8 eV can be fitted into the peaks at 710.6 eV and 708.4 eV. The fitted peaks at 723.7 eV and 710.6 eV are attributed to the Fe^3+^ 2p_1/2_ and Fe^3+^ 2p_3/2_ peaks, respectively, whereas the peaks at 721.7 eV and 708.4 eV correspond to the Fe^2+^ 2p_1/2_ and Fe^2+^ 2p_3/2_ peaks [33]. The area ratio of the peaks associated with Fe^3+^ to Fe^2+^ is approximately 2:1, consistent with the atomic molar ratio of Fe^3+^ to Fe^2+^ in Fe_3_O_4_, further confirming the complete reduction of Fe_2_O_3_ to Fe_3_O_4_ in the TiO_2_@Fe_2_O_3_ microspheres during the carbonization process, as supported by XRD results. Additionally, a satellite vibration peak is observed at 716.5 eV, indicating the existence of Fe_3_O_4_ [22,34].

Figure 3c displays the high-resolution XPS spectra of O 1s, revealing four distinct peaks following the fitting analysis. The peak at 530.4 eV corresponds to either Ti-O or Fe-O bonds, which often merge into a single peak due to their proximity, indicating the presence of oxides of titanium or iron. The peak at 531.5 eV corresponds to the Fe-O-C chemical bond, which may be formed by the in situ redox reaction between iron oxides and its external carbon during carbonization [35,36], implying that the carbon layer is tightly bonded to the Fe_3_O_4_, and that this tight bonding can inhibit the volumetric change in the Fe_3_O_4_ nanoparticles during charging and discharging [37,38]. Due to the low content of Fe in the material, the peak for Fe-O-C is relatively weak. Peaks at 532.3 and 533.7 eV correspond to C-O and C=O bonds, respectively [39]. The high-resolution XPS spectrum of C 1s, as shown in Figure 3d, features a peak at 284.8 eV associated with C-C bonds, while peaks at 286.0 and 288.7 eV correspond to C-O and C=O bonds, respectively [40,41], further validating the presence of a carbon layer on the surface.

### 3.2. Morphological Characterization

**Figure 4:**
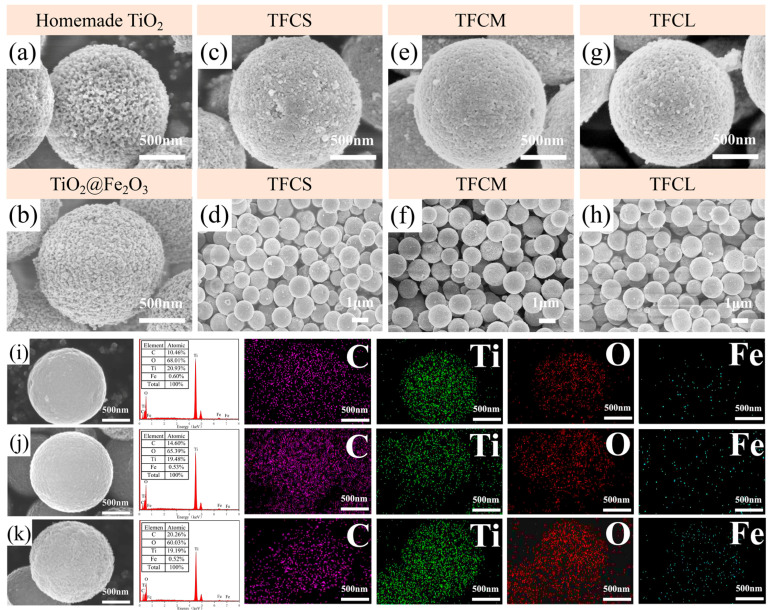
SEM images of the (**a**) TiO_2_, (**b**) TiO_2_@Fe_2_O_3_, (**c**,**d**) TFCS, (**e**,**f**) TFCM (**g**,**h**) TFCL; The EDX spectrum and EDS mapping images of the (**i**) TFCS, (**j**) TFCM, (**k**) TFCL.

Figure 4a and Figure 4b, respectively, display the field emission scanning electron microscopy (FE-SEM) images of homemade mesoporous TiO_2_ microspheres and TiO_2_@Fe_2_O_3_ microspheres. The images reveal that the microspheres have a diameter of approximately 1.5 μm, with rough surfaces and a multitude of mesopores, enhancing their specific surface area, which is favorable for the further coating of other materials on their surfaces. Figure 4c–h showcase the FE-SEM images of TFCS (c, d), TFCM (e, f), and TFCL (h, i) at different magnifications, illustrating that the microspheres, post-carbon-coating, retain good sphericity and dispersion. Their diameters do not exhibit significant change compared to the precursor TiO_2_ mesoporous microspheres and TiO_2_@Fe_2_O_3_ microspheres. However, the surfaces of the microspheres have become smoother and exhibit a noticeable change in texture compared to the precursor mesoporous TiO_2_ and TiO_2_@Fe_2_O_3_ microspheres. This observation leads us to conclude that the surfaces of these microspheres are indeed coated with a carbon layer, which is probably thin.

From Figure 4c–h, it is observable that the surfaces of the three types of TiO_2_@Fe_3_O_4_@C microspheres still retain porosity. When these microspheres serve as anode materials, the pores on their surfaces facilitate the penetration of the electrolyte into the interiors of the microspheres. This enhances the efficiency of lithium ions insertion/extraction, thereby improving the electrochemical performance of the batteries [42]. Figure 4i–k display the FE-SEM images of TFCS, TFCM, and TFCL microspheres alongside the X-ray energy dispersive spectroscopy (EDS) of C, Ti, Fe, and O elements within the field of view. The results show that C, Ti, Fe and O elements are present on the surface of the three microspheres and these four elements are uniformly distributed in the field of view, in which the proportion of carbon atoms is 10.46%, 14.60%, and 20.26% with the increase in the amount of PVP added, while the proportion of O, Ti, and Fe atoms decreases slightly. It is worth noting that the distribution of Fe elements in the TFCS, TFCM, and TFCL microspheres is relatively sparse, but a significant amount of Ti elements can be detected, suggesting that the carbon and iron oxide layers are thin, allowing the electron beam to pass through the coating layer of these two materials and scan the TiO_2_ inside.

The above results show that the TFCS, TFCM, and TFCL microspheres with different PVP additions are highly spherical and well dispersed, and all of them have a carbon layer encapsulated on the surface, and the carbon layer and the internal Fe_3_O_4_ layer are relatively thin.

### 3.3. Electrochemical Performance

**Figure 5:**
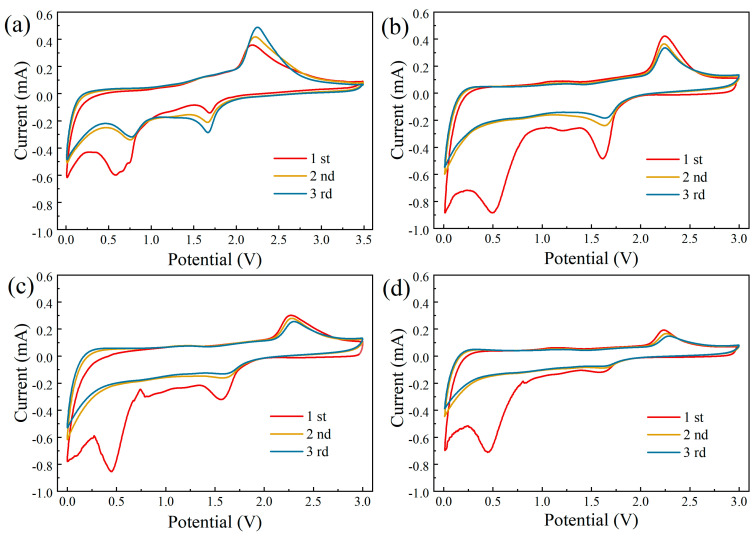
CV curves of (**a**) TiO_2_@Fe_2_O_3_ and (**b**) TFCS, (**c**) TFCM, and (**d**) TFCL microspheres at the scanning rate of 0.1 mV s^−1^ in the range of 0.01~3.5 V.

In order to study the effect of the coated carbon layer on the electrochemical performance of TiO_2_@Fe_2_O_3_, we fabricated half-cells with TiO_2_@Fe_2_O_3_, TFCS, TFCM, and TFCL as the anode materials (working electrodes), respectively, to test and compare their electrochemical performances. Figure 5a–d show the cyclic voltammetry curves of the four anode materials mentioned above for the initial three cycles at a scanning rate of 0.1 mV/s, respectively. It can be seen in the figures that the four materials have similar redox reactions in the first cycle, with the reduction peak first appearing at 1.7 V during the discharge process, which together with the oxidation peak at 2.3 V during the charging process corresponds to the characteristic peak of the insertion/extraction reaction of Li ions in anatase TiO_2_ [43], and the specific reaction is shown in Equation (1a). As can be seen in Figure 5a, a series of reduction peaks appeared in the range of 0.6 to 0.8 V in the first loop scan, which corresponds to the multi-step reaction of the reduction of Fe_2_O_3_ to Fe^0^ [44]. These peaks evolved into a reduction peak at 0.8 V during subsequent cycles, and it is hypothesized that the disappearing reduction peaks are due to the insertion of Li^+^ into irreversible sites and the generation of SEI (Solid Electrolyte Interface) membranes [14]. The reduction peak located at 0.8 V together with the broad oxidation peak in the range of 1.5–2.5 V that is not evident during charging corresponds to the reaction of Li^+^ insertion/extraction of Fe_2_O_3_. For TFCS, TFCM, and TFCL anode materials, as shown in Figure 5b–d, the reduction peak appearing at 0.4 V corresponds to the multi-step reaction of Fe_3_O_4_ reduction to Fe^0^ [45] as shown in Equations (1a,c). This peak transforms into an indistinct broad peak at 0.8 V during subsequent cycling, which is a phenomenon that may be attributed to the creation of the SEI film and the insertion of a large amount of Li^+^ into irreversible sites by the reaction of C with Li^+^ to form LiC_6_ [29,35]. This broad reduction peak and the inconspicuous broad oxidation peak in the range of 1.5 to 2.5 V during charging correspond to the reaction of Li^+^ insertion/extraction from Fe_3_O_4_ [46] as shown in Equation (1d). The Li^+^ insertion/extraction reaction of TiO_2_@Fe_3_O_4_@C microsphere anode materials can be represented by Equations (1a,d).

TiO_2_ + *x*Li^+^ + *x*e^−^ ↔ Li*_x_*TiO_2_ (1a)

Fe_3_O_4_ + 2Li^+^ + 2e^−^ ↔ Li_2_(Fe_3_O_4_) (1b)

Li_2_(Fe_3_O_4_) + 6Li^+^ + 6e^−^ ↔ 3Fe^0^ + 4Li_2_O (1c)

Fe_3_O_4_ + 8Li^+^ + 8e^−^ ↔ 3Fe^0^ + 4Li_2_O (1d)

In the subsequent two cycles, comparing the four materials, the CV curves of the three TiO_2_@Fe_3_O_4_@C microspheres have a higher degree of overlap, suggesting that the insertion/extraction reaction of Li ions has a better stability and reversibility in these materials. In addition, the oxidation and reduction peaks of Fe_3_O_4_ in the three TiO_2_@Fe_3_O_4_@C microspheres are relatively weaker, which can be attributed to the lower content of elemental Fe in the materials as well as the coating of carbon layer. The intensity of the oxidation and reduction peaks further decreases with the increase in the carbon coating of the materials corroborates the speculation. The CV curves of the three TiO_2_@Fe_3_O_4_@C microsphere materials show a high degree of consistency in morphology, indicating the same lithiation reaction properties, implying that the three TiO_2_@Fe_3_O_4_@C microsphere materials have similar compositions and structures, which is in agreement with the results of the analyses of their structures and morphologies above.

**Figure 6:**
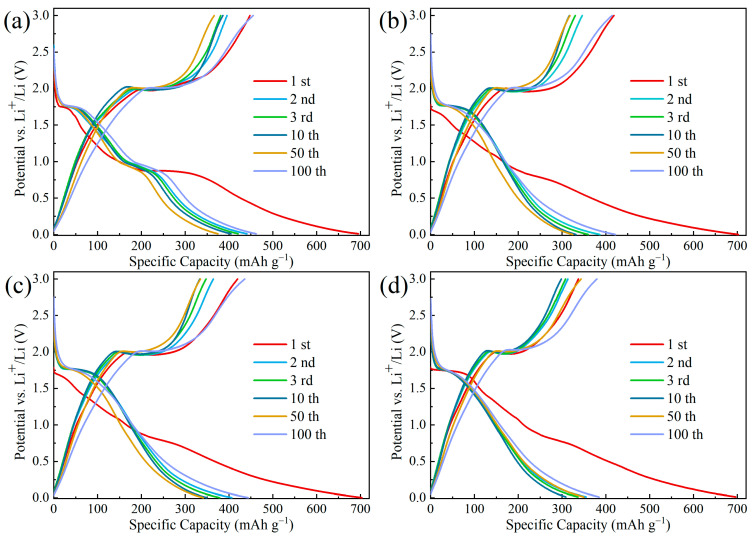
Galvanostatic discharge/charge curves for the 1st, 2nd, 3rd, 10th, and 100th cycles at a current density of 0.2 C for (**a**) TiO_2_@Fe_2_O_3_, (**b**) TFCS, (**c**) TFCM, and (**d**) TFCL microspheres.

Figure 6a–d shows the charge/discharge curves of TiO_2_@Fe_2_O_3_, TFCS, TFCM, and TFCL, respectively, at a current density of 0.2 C. The charge/discharge specific capacities of the 1st, 2nd, 3rd, 10th, 50th, and 100th cycles of these four anode materials are shown in Table 1. During the first discharge, a clear plateau at 1.75 V was observed for all four materials, which, together with the plateau at 2.0 V during charging, corresponds to the insertion/extraction of Li ions at the octahedral interstitial sites of anatase TiO_2_ [47,48]. As shown in Figure 6b, the specific capacity of TFCS microspheres at the first charge/discharge is 418.4/702.1 mAh·g^−1^, and its initial Coulombic efficiency is calculated to be 59.59%. Combined with the CV curves shown in Figure 5b, it is hypothesized that the larger specific capacity loss of the TFCS microspheres is attributed to the large amount of Li^+^ inserted in the irreversible sites as well as the generation of SEI membranes [29]. For TFCS microspheres, the charge/discharge curves do not change significantly during the subsequent cycles. The coulombic efficiencies of the 2nd, 3rd, 10th, 50th, and 100th cycles are 89.52%, 91.72%, 95.48%, 98.05%, and 98.15%, respectively. This proves that the Li^+^ insertion/extraction process of this anode material has good reversibility, and the lithiation reaction gradually tends to stabilize with the increase in the number of cycles. The charge/discharge curves of TFCM and TFCL are shown in Figure 6c and Figure 6d, respectively, and they are not significantly distinguished from Figure 6b, which, combined with the analysis of Figure 5b–d, may be due to the similarity of the lithiation reaction of these three materials. Comparing the charge-specific capacity (i.e., reversible capacity), it can be found that the charge-specific capacities of TFCS (414.2 mAh·g^−1^), TFCM (436.0 mAh·g^−1^), and TFCL (379.5 mAh·g^−1^) at the 100th cycle are slightly lower than that of the TiO_2_@Fe_2_O_3_ (455.2 mAh·g^−1^) cathode material. This may be attributed to the fact that the amorphous carbon layer coated on the surface basically does not provide lithium storage sites. Additionally, with the Fe_2_O_3_ in the TiO_2_@Fe_2_O_3_ reduced to Fe_3_O_4_, the theoretical specific capacity of Fe_3_O_4_ (926 mAh·g^−1^) is lower than that of Fe_2_O_3_ (1007 mAh·g^−1^). This further reduces the specific capacity of the material. Comparing the specific charge capacities of TFCS, TFCM, and TFCL at the 100th cycle, it is observed that TFCM exhibits a higher specific charge capacity than the other two materials, and the reason for the smallest specific charge capacity of TFCL is probably due to the fact that the amorphous carbon layer coated on the surface is too thick, which does not provide enough effective lithium storage sites. The larger charging capacity of TFCM compared to TFCS may be attributed to the fact that the carbon layer coated on the TFCS microspheres is too thin, which leads to the limited ability to resist the volume expansion of the material, resulting in the loss of capacity during the cycling process.

**Figure 7:**
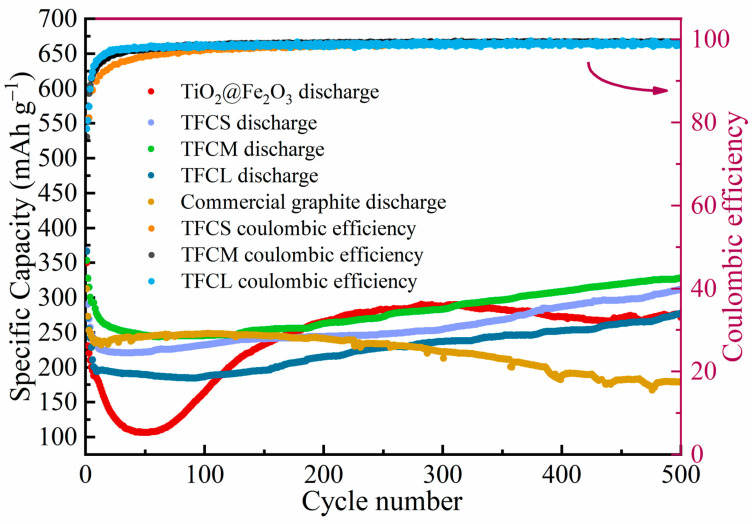
Cycling performances of TiO_2_@Fe_2_O_3_, TFCS, TFCM, TFCL, commercial graphite and coulombic efficiencies of TFCS, TFCM, TFCL at the current density of 2 C.

To further estimate the cycling performance of the materials, Figure 7 illustrates the discharge capacities of five types of anode materials—TiO_2_@Fe_2_O_3_, TFCS, TFCM, TFCL, and commercial graphite—over 500 cycles at a current density of 2 C along with the Coulombic efficiencies of TFCS, TFCM, and TFCL. In the initial 50 cycles, a noticeable decline in the discharge capacities of TiO_2_@Fe_2_O_3_, TFCS, TFCM, and TFCL is observed, which is possibly due to the internal reactions not having stabilized during the early stages of charging and discharging. Notably, the capacity decline for TFCS, TFCM, and TFCL is more gradual compared to the uncoated TiO_2_@Fe_2_O_3_ material, which is possibly because the amorphous carbon layer on the material surfaces reduces the detrimental effects of internal material volume expansion. Between the 50th and 300th cycles, the capacities of TFCS, TFCM, TFCL, and TiO_2_@Fe_2_O_3_ increase to different extents, which aligns with the findings shown in Figure 6a–d. From the 300th to the 500th cycle, the discharge capacity of TiO_2_@Fe_2_O_3_ shows a tendency to stabilize and decrease, whereas TFCS, TFCM, and TFCL continue to show stable capacity growth, indicating that the cycling stability of the materials is significantly improved by coating the microspheres with a carbon layer. The discharge capacities of TiO_2_@Fe_2_O_3_, TFCS, TFCM, TFCL, and commercial graphite at the 500th cycle are 271.9, 305.7, 328.3, 277.5, and 178.7 mAh·g^−1^, respectively. The first four materials exhibit superior performance compared to the commercial graphite anode, indicating a synergistic effect between their different components. The increased discharge capacities of TFCS, TFCM, and TFCL in comparison to TiO_2_@Fe_2_O_3_ can be attributed to the presence of an amorphous carbon layer that effectively reduces the decline of capacity caused by the volume expansion of iron oxides during the process of charging and discharging. Additionally, the incorporation of the carbon layer improves the electrical conductivity of the material, enabling higher capacity retention under high current. After 500 cycles, the Coulombic efficiencies of TFCS, TFCM, and TFCL all exceed 99.5%, demonstrating good charge–discharge reversibility. It is important to mention that among the three anode materials, TFCS, TFCM, and TFCL, TFCM exhibits the highest discharge specific capacity in the 500th cycle due to reasons similar to the analysis of the results in Figure 6b–d. Specifically, a carbon layer that is excessively thick cannot offer enough effective lithium storage sites, while a carbon layer that is excessively thin cannot withstand the expansion of the material volume.

**Figure 8:**
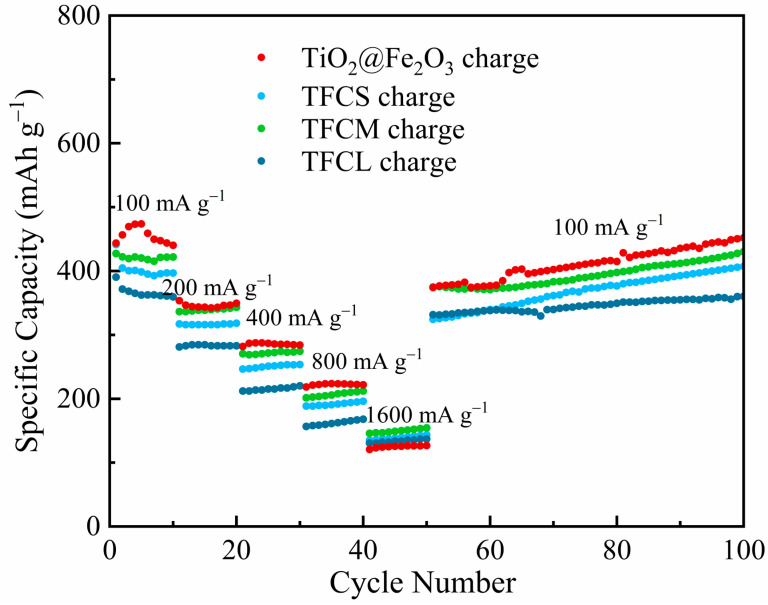
Rate performances of TiO_2_@Fe_2_O_3_, TFCS, TFCM and TFCL at the current densities of 100, 200, 400, 800, and 1600 mA g^−1^.

We further evaluated the electrochemical performance of materials under different current densities through rate capability tests. Figure 8 and Table 2 display the charge capacities (i.e., reversible capacities) of TiO_2_@Fe_2_O_3_, TFCS, TFCM, and TFCL at current densities of 100, 200, 400, 800, and 1600 mA·g^−1^. The results indicate that at a current density of 100 mA·g^−1^, the charge capacities of TFCS, TFCM, and TFCL are lower than that of TiO_2_@Fe_2_O_3_. However, as the current density increases, the charge capacities of TFCS, TFCM, and TFCL exceed that of TiO_2_@Fe_2_O_3_ at 1600 mA·g^−1^. This is because the surface carbon layer imparts high conductivity to the materials. The calculated capacity retention rates (i.e., the ratio of charge capacity at 100 mA·g^−1^ to that at 1600 mA·g^−1^) for TiO_2_@Fe_2_O_3_, TFCS, TFCM, and TFCL are 27.2%, 35.2%, 35.9%, and 36.9%, respectively, showing that the carbon-coated materials have nearly uniform and significantly superior capacity retention rates compared to TiO_2_@Fe_2_O_3_. This is primarily due to the enhanced electrical conductivity provided by the carbon layer, which in turn improves the rate performance of the materials, aligning with the analysis presented in Figure 7. Post-rate capability testing, the re-test results at a current density of 100 mA·g^−1^ show no significant change compared to the initial tests, indicating that high-current charge–discharge cycles have not caused significant damage to the structure of the materials.

**Figure 9:**
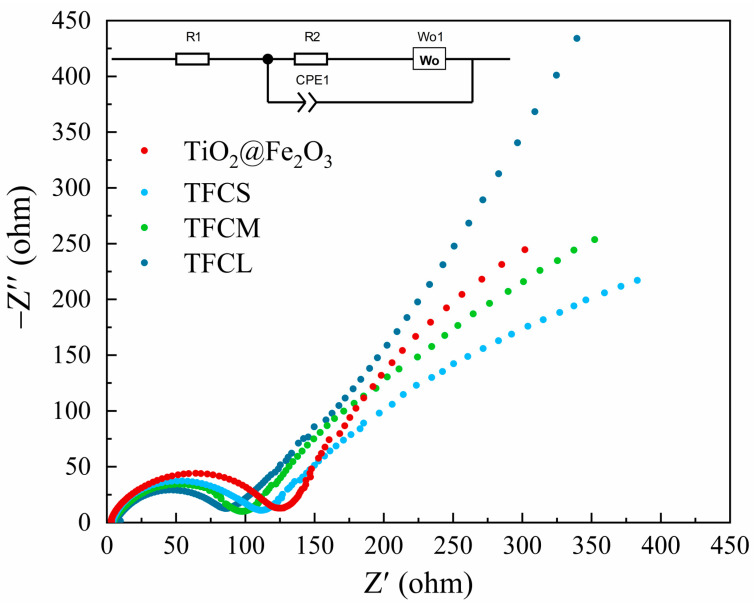
Nyquist plots of TiO_2_@Fe_2_O_3_, TFCS, TFCM and TFCL in the frequency range from 1 × 10^6^ to 0.01 Hz. The inset shows the corresponding equivalent circuit.

To gain a deeper insight into the influence of the carbon coating layer on the electronic conductivity and lithium-ion diffusion rate, we performed electrochemical impedance spectroscopy (EIS) tests on four anode materials: TiO_2_@Fe_2_O_3_, TFCS, TFCM, and TFCL. The resulting data are presented in Figure 9. Based on the lithium-ion insertion/extraction mechanism in the anode materials, the electrochemical impedance spectra can be fitted using an equivalent circuit (shown in the inset of Figure 9). In this model, CPE1 represents the double-layer capacitance, R1 indicates the internal resistance of the battery, R2 signifies the charge transfer resistance, and Wo1 represents the Warburg coefficient for lithium-ion diffusion [43,49]. The fitting results are shown in Table 3. The internal resistances for the TiO_2_@Fe_2_O_3_, TFCS, TFCM, and TFCL anode materials are 2.08, 4.03, 4.82, and 6.25 Ω, respectively, while the charge transfer resistances are 119.8, 98, 94.42, and 81.92 Ω, respectively, reflecting the conductivity of the materials. It is evident that the conductivity of TFCS, TFCM, and TFCL is significantly higher than that of TiO_2_@Fe_2_O_3_. Furthermore, comparing TFCS, TFCM, and TFCL materials, the impedance decreases with the increasing thickness of the amorphous carbon coating layer, indicating that the carbon layer significantly enhances the material’s conductivity. This enhancement is likely due to two reasons: (i) the amorphous carbon layer itself has a high conductivity; (ii) the coated carbon promotes the reduction of Fe_2_O_3_ to Fe_3_O_4_, which exhibits better conductivity due to the rapid electron transfer between Fe^2+^ and Fe^3+^. These results demonstrate that the carbon-coated TFCS, TFCM, and TFCL anode materials possess good conductivity, which is one of the reasons for their excellent capacity retention under high current density charge and discharge conditions, and this is consistent with the results shown in Figure 8 and Figure 9.

## 4. Conclusions

In this study, a homogeneous precipitation was utilized to deposit a layer of Fe_2_O_3_ on the surface of homemade TiO_2_ microspheres, resulting in the formation of TiO_2_@Fe_2_O_3_ microspheres. Subsequently, PVP molecules were adsorbed onto these microspheres, and high-temperature carbonization converted the adsorbed PVP molecules into a carbon layer. During this process, some Fe^3+^ ions were reduced to Fe^2+^, ultimately producing three types of double-layer core–shell structured TiO_2_@Fe_3_O_4_@C microspheres: TFCS, TFCM, and TFCL, with increasing amounts of carbon coating on the outer layer, respectively. The structure of the TiO_2_@Fe_3_O_4_@C microspheres was investigated using XRD, Raman, and XPS techniques, while their micromorphology was characterized by FE-SEM. The study also explored the effect of the carbon coating amount on the electrochemical performance of the TiO_2_@Fe_3_O_4_@C microspheres. The following conclusions were drawn:

(i) Results from XRD, Raman, and XPS analyses indicate that during the preparation of TiO_2_@Fe_3_O_4_@C microspheres, the relatively low content of Fe_2_O_3_ in TiO_2_@Fe_2_O_3_ microspheres was completely reduced to Fe_3_O_4_. The TFCS, TFCM, and TFCL microspheres are all composed of anatase TiO_2_, magnetite Fe_3_O_4_, and an amorphous carbon layer on the surface.
(ii) EDS results reveal the presence of C, Ti, Fe, and O elements in the TFCS, TFCM, and TFCL microspheres. FE-SEM images show that the TFCS, TFCM, and TFCL microspheres have high sphericity and good dispersibility with a carbon layer coating the surface.
(iii) At a current density of 2 C, after 500 cycles, the specific capacities of TiO_2_@Fe_2_O_3_, TFCS, TFCM, and TFCL are 271.9, 305.7, 328.3, and 277.5 mAh·g^−1^, respectively, which are all significantly higher than commercial graphite (178.7 mAh·g^−1^). The specific capacities of TFCS, TFCM, and TFCL are higher than that of TiO_2_@Fe_2_O_3_, which is attributed to the fact that the coated amorphous carbon layer can effectively slow down the capacity decay caused by the volume change in iron oxides during charging and discharging. Among the three anode materials, TFCM exhibited the highest specific capacity, benefitting from its suitable thickness of its carbon coating layer.
(iv) In terms of cycle stability, TFCS, TFCM, and TFCL significantly outperform TiO_2_@Fe_2_O_3_. After 300 cycles at a current density of 2 C, the specific capacities of TFCS, TFCM, and TFCL continued to show stable growth.
(v) Within the range of 100 to 1600 mA·g^−1^, the capacity retention rates of TiO_2_@Fe_2_O_3_, TFCS, TFCM, and TFCL are 27.2%, 35.2%, 35.9%, and 36.9%, respectively. The carbon coating layer enhanced the electrical conductivity of the TiO_2_@Fe_3_O_4_@C materials, thereby improving their rate performance.

This study provides an insight into the modification and carbon-coating strategy of anode materials for lithium-ion batteries. The abundance and environmental friendliness of iron, titanium, and carbon resources in the TiO_2_@Fe_3_O_4_@C microsphere anode material give this strategy a cost advantage. The enhanced cycle life of this anode material expands the application range of lithium-ion batteries in electric vehicles and portable energy storage devices, enabling them to meet more diverse usage conditions and stricter performance requirements.

## Figures and Tables

**Table 1:** Charge/discharge specific capacities (mAh g^−1^) of TiO_2_@Fe_2_O_3_, TFCS, TFCM, and TFCL anode materials at 0.2 C current density for the 1st, 2nd, 3rd, 10th, 50th, and 100th cycles.

Materials	Cycling Numbers (Cycles)					
	1	2	3	10	50	100
TiO_2_@Fe_2_O_3_	447.7/695.5	395.2/440.8	381/421.2	386.1/404.4	366.2/374.7	455.2/462.1
TFCS	418.4/702.1	346.1/386.6	330.2/360.1	316.9/331.9	318.1/324.4	414.2/422
TFCM	419.5/703.9	364.3/406.9	347.6/379	333.5/349.3	334.9/341.5	436/444.2
TFCL	337.4/696.7	313.4/355	308.1/336.6	298.5/308.9	343.4/349.5	379.5/384.6

**Table 2:** Charge-specific capacities (reversible capacities) (mAh g^−1^) of TiO_2_@Fe_2_O_3_, TFCS, TFCM, and TFCL anode materials at current densities of 100, 200, 400, 800, 1600, and 100 mA g^−1^ (after).

Materials	Current Densities (mA g^−1^)					
	100	200	400	800	1600	100 (after)
TiO_2_@Fe_2_O_3_	462.2	342.8	285.9	223.6	125.6	411.2
TFCS	395.3	316.1	251.4	191.8	139.3	373.4
TFCM	417.9	339.6	272.3	207.9	150	391.4
TFCL	362.7	282.9	215.4	162.5	133.8	347.1

**Table 3:** R1 (ohmic resistance) and R2 (charge transfer impedance) values obtained from equivalent circuit fitting for TiO_2_@Fe_2_O_3_, TFCS, TFCM and TFCL anode materials in LIBs.

Materials	R1	R2
TiO_2_@Fe_2_O_3_	2.08	119.81
TFCS	4.03	98.00
TFCM	4.82	94.42
TFCL	6.25	81.92

## Data Availability

Data are contained within the article.
